# Identifying Diagnostic MicroRNAs and Investigating Their Biological Implications in Rectal Cancer

**DOI:** 10.1001/jamanetworkopen.2021.36913

**Published:** 2021-12-03

**Authors:** Jin K. Kim, Xuan Qu, Chin-Tung Chen, J. Joshua Smith, Francisco Sanchez-Vega, Julio Garcia-Aguilar

**Affiliations:** 1 Department of Surgery, Colorectal Service, Memorial Sloan Kettering Cancer Center, New York; 2 Department of Epidemiology and Biostatistics, Memorial Sloan Kettering Cancer Center, New York

## Abstract

**Importance:**

Accurate clinical staging is important in rectal cancer because it determines the appropriate treatment and prognosis. Despite the use of multiple diagnostic imaging tools, it is sometimes difficult to clinically distinguish stage I tumors from stage II or III locally advanced disease. Identification of differentiating microRNAs (miRNAs) between these 2 groups may improve the clinical diagnostic power and provide insight into the biology of tumor progression.

**Objectives:**

To investigate differences in the expression of miRNAs in stage I vs stage II or III rectal cancers and integrate matched mRNA profiling data to identify possible functional roles of these miRNAs.

**Design, Setting, and Participants:**

The primary tumor specimens from patients who were enrolled in 2 prospective clinical trials between March 24, 2004, and November 16, 2012 (American College of Surgeons Oncology Group [ACOSOG] Z6041 and Timing of Rectal Cancer Response to Chemoradiation [TIMING]) were sequenced to arrive at a set of 127 cases (41 stage I and 86 stage II or III tumors) with matched miRNA and messenger RNA (mRNA) profiling data. These findings were also evaluated in an independent cohort of 127 patient specimens (29 stage I and 98 stage II or III tumors) from The Cancer Genome Atlas Rectum Adenocarcinoma (TCGA-READ) that also had matched miRNA and mRNA data. Data analysis was performed from September 1, 2019, to September 1, 2020.

**Main Outcomes and Measures:**

Alterations in miRNA expression between stage I and stage II or III tumors and their potential gene targets.

**Results:**

A total of 254 pretreatment rectal adenocarcinoma specimens were analyzed in this study as 2 distinct cohorts: 127 samples in the ACOSOG/TIMING (stage I group: 27 [66%] male; mean [SD] age, 64.4 [10.8] years; stage II or III group: 47 [55%] male; mean [SD] age, 57.0 [11.4] years), and another 127 samples from TCGA-READ (stage I group: 17 [59%] male; mean [SD] age, 63.6 [12.0] years; stage II or III group: 48 [49%] male; mean [SD] age, 64.5 [11.4] years). A total of 19 miRNAs were overexpressed in stage II or III vs stage I tumors in both cohorts. This miRNA signature had an excellent discriminative value for distinguishing stage II or III from stage I rectal tumors (area under the curve, 0.88; 95% CI, 0.83-0.94 in ACOSOG/TIMING cohort and area under the curve, 0.84; 95% CI, 0.77-0.91 in the TCGA-READ cohort). Integrative analysis revealed 3 miRNA-mRNA pairs that exhibited significant correlations in both cohorts: miR-31-5p-*SATB2*, miR-143-3p-*KLF5*, and miR-204-5p-*EZR*.

**Conclusions and Relevance:**

This diagnostic study found that many of the dysregulated miRNAs in stage II or III vs stage I rectal cancers have biological implications for tumor progression. The results of this study suggest that these miRNAs could assist as diagnostic biomarkers to better identify patients with locally advanced rectal cancer.

## Key Points

**Question:**

Can the expression levels of microRNA (miRNA) in the primary tumor distinguish locally advanced disease among nonmetastatic rectal cancers?

**Findings:**

This diagnostic study of 2 independent cohorts found that a set of 19 miRNAs can effectively distinguish locally advanced disease (stage II or III) among nonmetastatic rectal cancers. Within this set of miRNAs, 3 miRNA-mRNA functional interactions were identified by integrative transcriptome analysis.

**Meaning:**

These results suggest that the identified miRNAs in this study have potential to serve as diagnostic biomarkers in rectal cancer.

## Teaser

This diagnostic study explores the diagnostic accuracy of differentially expressed miRNAs between stage I and stage II or III rectal cancers.

## Introduction

The current standard of care for stage II or III rectal cancer involves multimodal therapy, including preoperative chemoradiation, surgery, and chemotherapy.^1^ Patients with stage I disease, on the other hand, can often be treated with surgery alone.^1^ A variety of diagnostic tools are used to clinically differentiate the stage of these tumors, but the accuracy is limited.^2,3^ Supplementation with a reliable biomarker may help overcome this current limitation.

MicroRNAs (miRNAs) are 19- to 25-nucleotide, long, noncoding RNAs that play important roles in many biological processes that involve the cell cycle and cellular differentiation.^4,5^ In colorectal cancer, miRNAs are dysregulated and have diverse roles in tumor biology, such as angiogenesis and proliferation and invasion of cancer cells.^5^ Several miRNAs have already been identified as potential biomarkers for diagnosis and prognosis in patients with colorectal cancer.^6^ Functionally, miRNAs negatively regulate the gene expression of their target mRNA at the posttranscriptional level.^4^ MicroRNA is initially folded into a precursor hairpin (pre-miRNA) and then cleaved to form an RNA duplex.^4^ Afterward, the RNA-induced silencing complex unwinds the duplex into 2 single strands.^4^ Usually, 1 of the 2 single-strand miRNAs is quickly degraded; however, this process is dysregulated in cancer.^7^ Both complementary strands of the mature miRNA play roles in cancer and sometimes in ways that oppose each other.^7^ Moreover, there are many combinations of miRNA and mRNA interactions because 1 miRNA can have multiple mRNA targets and 1 gene can be targeted by multiple miRNAs.

Many groups have found that miRNA expression profiles are associated with the clinical stage of colorectal cancer.^8,9^ Some have also used miRNA to predict potential interactions with relevant oncogenic pathways.^8,10,11^ In this study, we analyzed 2 independent patient cohorts to identify differentially expressed miRNAs between stage I and stage II or III rectal cancers and explore the diagnostic accuracy of this miRNA signature. We then integrated matched mRNA sequencing data to investigate potential miRNA-mRNA interactions that are associated with the clinical stage to gain insight into the biological functions of these miRNAs.

## Methods

### Patients

A total of 127 pretreatment rectal adenocarcinoma endoscopic biopsy samples from 2 prospective multicenter clinical trials formed the American College of Surgeons Oncology Group (ACOSOG)/Timing of Rectal Cancer Response to Chemoradiation (TIMING) cohort. A total of 41 biopsy specimens from patients with American Joint Committee on Cancer stage I tumors from the ACOSOG-Z6041 trial^12^ and 86 biopsy specimens from patients with stage II or III tumors from the ACOSOG/TIMING trial^13^ were assessed. The ACOSOG-Z6041 trial was a phase 2, nonrandomized trial that enrolled patients with clinical T2N0 tumors measuring less than 4 cm in greatest diameter and located within 8 cm of the anal verge from May 25, 2006, to October 22, 2009. The TIMING trial was a phase 2, nonrandomized trial that enrolled patients from March 24, 2004, to November 16, 2012. Patients in this study had clinical stage II or III disease located within 12 cm of the anal verge. The median follow-up of these patients was 54 months (IQR, 46-63 months) for ACOSOG-Z6041 and 59 months (IQR, 48-65 months) for ACOSOG/TIMING. Patients in both clinical trials provided written informed consent for research using biospecimens. All data were deidentified. Data analysis was performed from September 1, 2019, to September 1, 2020. A central institutional review board and the institutional review boards at each participating institution approved the study. This study followed the Standards for Reporting of Diagnostic Accuracy (STARD) reporting guideline.

An independent cohort of patient sequencing data of miRNA and mRNA from 127 patients with rectal adenocarcinoma (29 with stage I tumors and 98 with stage II or III tumors) was obtained from The Cancer Genome Atlas (TCGA) to validate our findings. This is a publicly available database that does not require institutional review board approval.

### Patient Sample Processing

Biopsy specimens were preserved as formalin-fixed, paraffin-embedded (FFPE) tissue blocks. Tumor enriched areas (>70% of tissue area) were marked on hematoxylin-eosin–stained slides by a pathologist at each participating site. Ten 10-mm-thick unstained slides were then cut, and the marked areas were macrodissected guided by the hematoxylin-eosin–stained slides.

### Nucleic Acid Extraction and Sequencing

DNA and RNA were extracted using AllPrep DNA/RNA FFPE kits (Qiagen). Total RNA was amplified to generate complementary DNA libraries using the Ovation FFPE WTA System (NuGEN Technologies) and sent for Affymetrix U133 Plus 2.0 Array (Affymetrix). Total RNA was also used to generate libraries for miRNA deep sequencing using the HiSeq 2000 Platform (Illumina). *KRAS* (OMIM 190070) mutations were determined by standard polymerase chain reaction followed by Sanger sequencing of exons 2 and 3.

### Computational Processing of miRNA Data of the ACOSOG/TIMING Cohort

After raw reads were extracted from FASTQ files, the 3′ adaptor sequences ATCTCGTATGCCGTCTTCTGCTTG were cleaned up by using Cutadapt (National Bioinformatics Infrastructure Sweden). Processed reads with a length less than 8 were discarded. The sequences were collapsed using seqcluster to apply filters based on abundances and shorten computational time.^14,15^ We then converted the FASTQ files to FASTA format and performed the miRNA alignment with the tool miraligner. The raw miRNA expression matrix was extracted after the annotation using package isomiRs, version 1.16.2 in R software (R Foundation for Statistical Computing).^15^

### Normalization

We applied a regularized log transformation to the raw miRNA expression matrix in our cohort from package DESeq2, version 1.28.1 in R.^16^ As for the microarray data, a Robust Multichip Average algorithm from the package oligo was used for normalization.^17^

A variance stabilizing transformation from the package DESeq2 was applied to the miRNA data in TCGA Rectum Adenocarcinoma (TCGA-READ). Condition quantile normalization from the package cqn was used to transform the mRNA data in TCGA-READ. This algorithm combines robust generalized regression to correct systematic biases.^18^

### Statistical Analysis

Differential expression analysis between stage I and stage II or III tumors was conducted with the package DESeq2 in R, which uses a Wald test.^16^ A total of 1426 miRNAs were investigated. We used the false discovery rate (FDR) to correct for multiple hypothesis testing.^19^ In the ACOSOG/TIMING cohort, miRNAs with an FDR less than 0.05 and an absolute value of log_2_ fold change greater than log_2_(1.5) were considered differentially expressed in our cohort. In the TCGA-READ cohort, miRNAs with a 2-sided *P* < .05 that were expressed in the same direction between stage I and stage II or III tumors as observed in the ACOSOG/TIMING cohort were selected.

We used binary logistic regression analysis to assess the discriminative power of our proposed 19 miRNA signature. This analysis was performed using the glm package, version 3.6.2 in R with the family of binomial choice for the outcome distribution. Receiver operating characteristic (ROC) curves were drawn using the empirical method from the ROCit package, version 2.1.1 in R. Empirical distributions of area under the curve (AUC) values based on the null hypothesis of the coefficients not being predictive were generated using random permutations, and a 2-sided *P* < .05 was considered significant. Calibration curves were drawn using the calibrate function from the rms package, version 6.2.0 in R. The val.prob function from the same package was used to compute the intercept and the slope of the calibration curves as well as the Brier score (mean squared difference between observed and predicted probabilities).

We computed the Spearman correlation to compare the normalized miRNA expression and corresponding mRNA expression data. *P* values associated with individual Spearman correlation coefficients were computed using the cor.test in R. All miRNA-mRNA pairs with a Spearman ρ less than 0 and an FDR less than 0.1 were considered significant. Then we visualized the significant miRNA-mRNA pairs in both cohorts with consistent enrichment direction.

All the targets of differentially expressed miRNAs in our cohort were identified using miRTarBase, version 8.0 (Institute of Bioinformatics and Systems Biology). The precursor miRNA names in the TCGA-READ cohort were converted to the mature miRNA names because the input requires the mature form of miRNA.

## Results

### Patient Characteristics

A total of 254 pretreatment rectal adenocarcinoma specimens were analyzed in this study as 2 distinct cohorts. A total of 127 samples were collected from ACOSOG/TIMING^12,20^ (stage I group: 27 [66%] male; mean [SD] age, 64.4 [10.8] years; stage II or III group: 47 [55%] male; mean [SD] age, 57.0 [11.4] years), and another 127 samples were from TCGA-READ^21,22^ (stage I group: 17 [59%] male; mean [SD] age, 63.6 [12.0] years; stage II or III group: 48 [49%] male; mean [SD] age, 64.5 [11.4] years). Because the treatment of patients with stage II and stage III rectal cancer is identical, we decided to group the 2 stages to compare against stage I tumors. Moreover, the clinical distinction of stage II from stage III disease is inaccurate,^2,3^ which further justifies our groupings. Matching sets of miRNA and mRNA were sequenced per patient and analyzed.

The demographic characteristics of the ACOSOG/TIMING and TCGA-READ cohorts are summarized in Table 1 and detailed in eTable 1 in the Supplement. Data on race and ethnicity were incomplete. *KRAS* was altered more frequently in the stage II or III tumors (45%) vs stage I tumors (26%; *P* = .049). Because the patient selection criteria that involved tumor size and tumor location were inherently different for the ACOSOG-Z6041 and TIMING trials, these differences were not statistically compared.

**Table 1.** Demographic Characteristics of the Patients in the Study Cohorts^a^

Characteristic	ACOSOG/TIMING cohort		TCGA-READ cohort	
	Stage I	Stage II or III	Stage I	Stage II or III
No. (%) of patients	41 (32)	86 (68)	29 (23)	98 (77)
Patient age, mean (SD), y	64.4 (10.8)	57.0 (11.4)	63.3 (12.0)	64.5 (11.4)
Sex				
Male	27 (66)	47 (55)	17 (59)	48 (49)
Female	14 (34)	39 (45)	12 (41)	50 (51)
Tumor distance from anal verge, mean (SD), cm	4.7 (1.8)	6.7 (3.3)	NA	NA
Tumor size, mean (SD), cm	3.0 (0.8)	4.9 (1.9)	NA	NA
cT class				
T1 or 2	41 (100)	11 (13)	29 (100)	4 (4)
T3	0	73 (85)	0	87 (89)
T4	0	2 (2)	0	7 (7)
cN class				
Negative	41 (100)	18 (21)	41 (100)	48 (49)
Positive	0	68 (79)	0	50 (51)

Abbreviations: ACOSOG/TIMING, American College of Surgeons Oncology Group/Timing of Rectal Cancer Response to Chemoradiation; NA, not applicable; TCGA-READ, The Cancer Genome Atlas Rectum Adenocarcinoma.

^a^ Data are presented as number (percentage) of patients unless otherwise indicated.

### Differential Expression of miRNAs in Stage I vs Stage II or III Rectal Cancer

**Figure 1.**
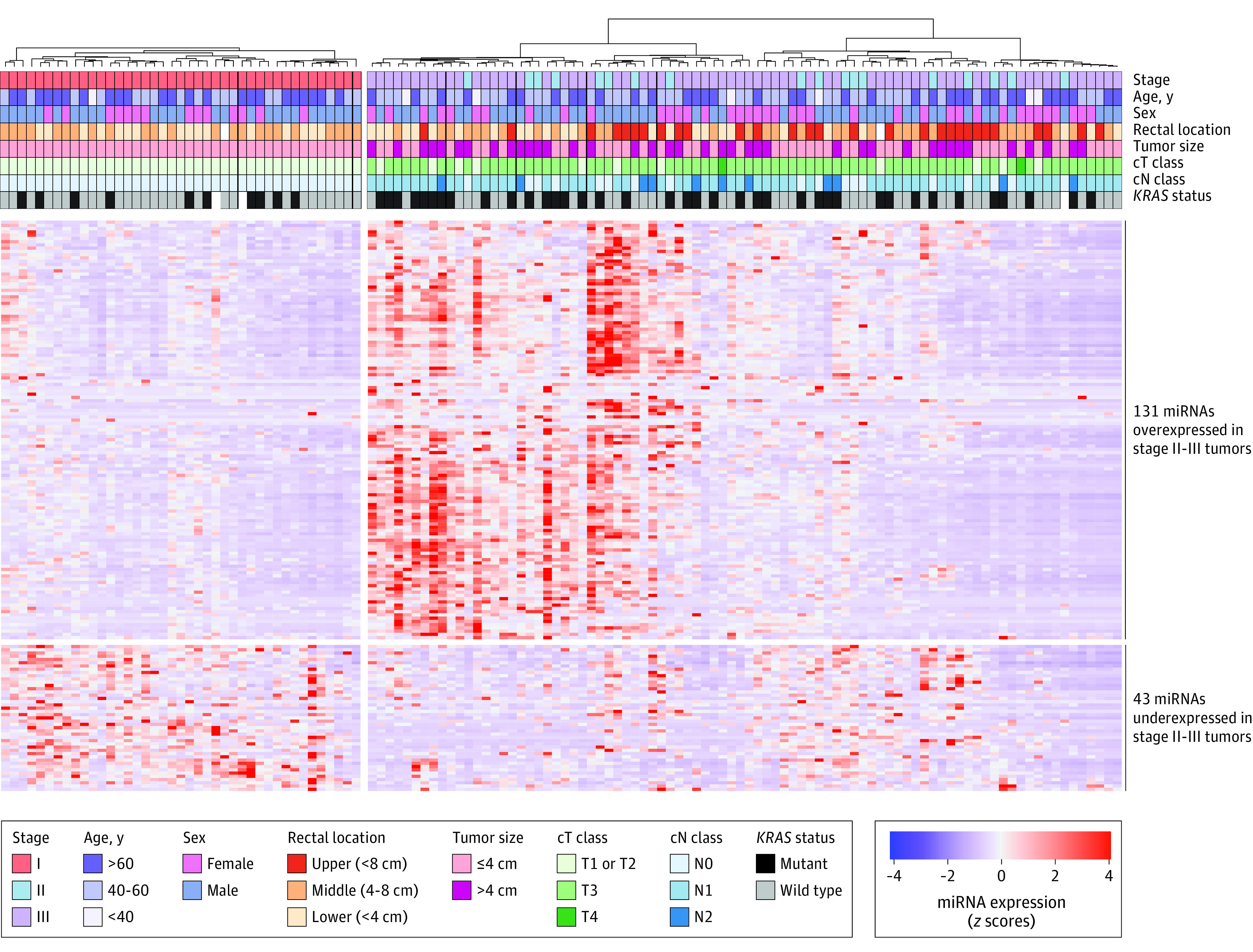
Differentially Expressed MicroRNAs (miRNAs) in Stage I vs Stage II or III Rectal Cancers in the American College of Surgeons Oncology Group/Timing of Rectal Cancer Response to Chemoradiation Cohort Heatmap of 174 differentially expressed miRNAs (log_2_ fold change >1.5; false discovery rate <0.05) in stage I vs stage II or III rectal cancers.

We examined whether the expression of miRNA was dysregulated in stage I vs stage II or III rectal cancers. The normalized miRNA expression matrix is provided in eTable 2 in the Supplement. We performed a differential expression analysis of the miRNA and found 174 miRNAs that met our cutoff. A total of 43 miRNAs were overexpressed in stage I vs II or III tumors, and 131 miRNAs were underexpressed in stage I vs stage II or III tumors (Figure 1; eTable 3 in the Supplement).

To evaluate our findings using an independent cohort, we used miRNA data from TCGA-READ.^21,22^ The normalized miRNA expression matrix is provided in eTable 4 in the Supplement. We performed differential expression analysis of the miRNA in stage I vs stage II or III tumors of the TCGA-READ cohort. The miRNAs that were differentially expressed in a pattern that was consistent with the ACOSOG/TIMING cohort were selected and filtered for *P* < .05 (eTable 5 in the Supplement). We found 19 miRNAs that were overexpressed in stage II or III tumors in both cohorts (Table 2). However, none of the miRNAs that were overexpressed in stage I vs II or III tumors in our ACOSOG/TIMING cohort exhibited significant overexpression in stage I vs II or III tumors within the TCGA-READ cohort. Because age was unevenly distributed across stage, we investigated correlations between age and expression of the 19 miRNAs but found no significant associations (eFigure 1A in the Supplement). We also compared computationally inferred levels of tumor purity across stage groups in the TCGA-READ cohort and discarded tumor cellularity as a confounding factor (eFigure 1B in the Supplement).

**Table 2.** miRNAs Overexpressed in Stage II or III vs Stage I Rectal Cancers Across 2 Independent Cohorts

miRNA	ACOSOG/TIMING		TCGA-READ^a^	
	Fold change	*Q* value	Fold change	*P* value
miR-99a-5p	1.81	5.68 × 10^4^	1.49	3.27 × 10^2^
miR-99a-3p	1.66	9.65 × 10^3^		
miR-34c-5p	2.36	2.43 × 10^6^	2.06	7.75 × 10^6^
miR-34c-3p	2.53	1.54 × 10^2^		
miR-31-5p	2.77	5.98 × 10^4^	1.76	3.96 × 10^2^
miR-31-3p	4.59	5.23 × 10^4^		
miR-218-5p	1.91	9.55 × 10^4^	1.6	9.40 × 10^4^
miR-214-5p	1.84	2.23 × 10^6^	1.29	4.40 × 10^2^
miR-214-3p	2.84	2.12 × 10^8^		
miR-204-5p	2.27	4.73 × 10^2^	2.55	1.96 × 10^3^
miR-145-5p	1.91	1.93 × 10^5^	1.79	3.35 × 10^4^
miR-143-5p	1.68	7.92 × 10^6^	1.4	2.51 × 10^2^
miR-143-3p	1.76	4.44 × 10^5^		
miR-133a-3p	1.65	1.11 × 10^2^	2.28	1.32 × 10^4^
miR-129-2-3p	3.12	8.55 × 10^3^	1.75	2.07 × 10^2^
miR-100-3p	2.43	8.47 × 10^3^	1.43	2.11 × 10^2^
let-7g-3p	1.58	4.64 × 10^2^	1.35	8.10 × 10^4^
let-7f-1-3p	1.83	3.24 × 10^4^	1.81	3.87 × 10^3^
let-7e-3p	1.65	6.27 × 10^4^	1.92	2.38 × 10^5^

Abbreviations: ACOSOG/TIMING, American College of Surgeons Oncology Group/Timing of Rectal Cancer Response to Chemoradiation; miRNA, microRNA; TCGA-READ, The Cancer Genome Atlas Rectum Adenocarcinoma.

^a^ The miRNA notation for TCGA-READ was converted to match the nomenclature for the mature sequences.

### Discrimination of miRNA Signatures in Early- and Late-Stage Rectal Cancers

**Figure 2.**
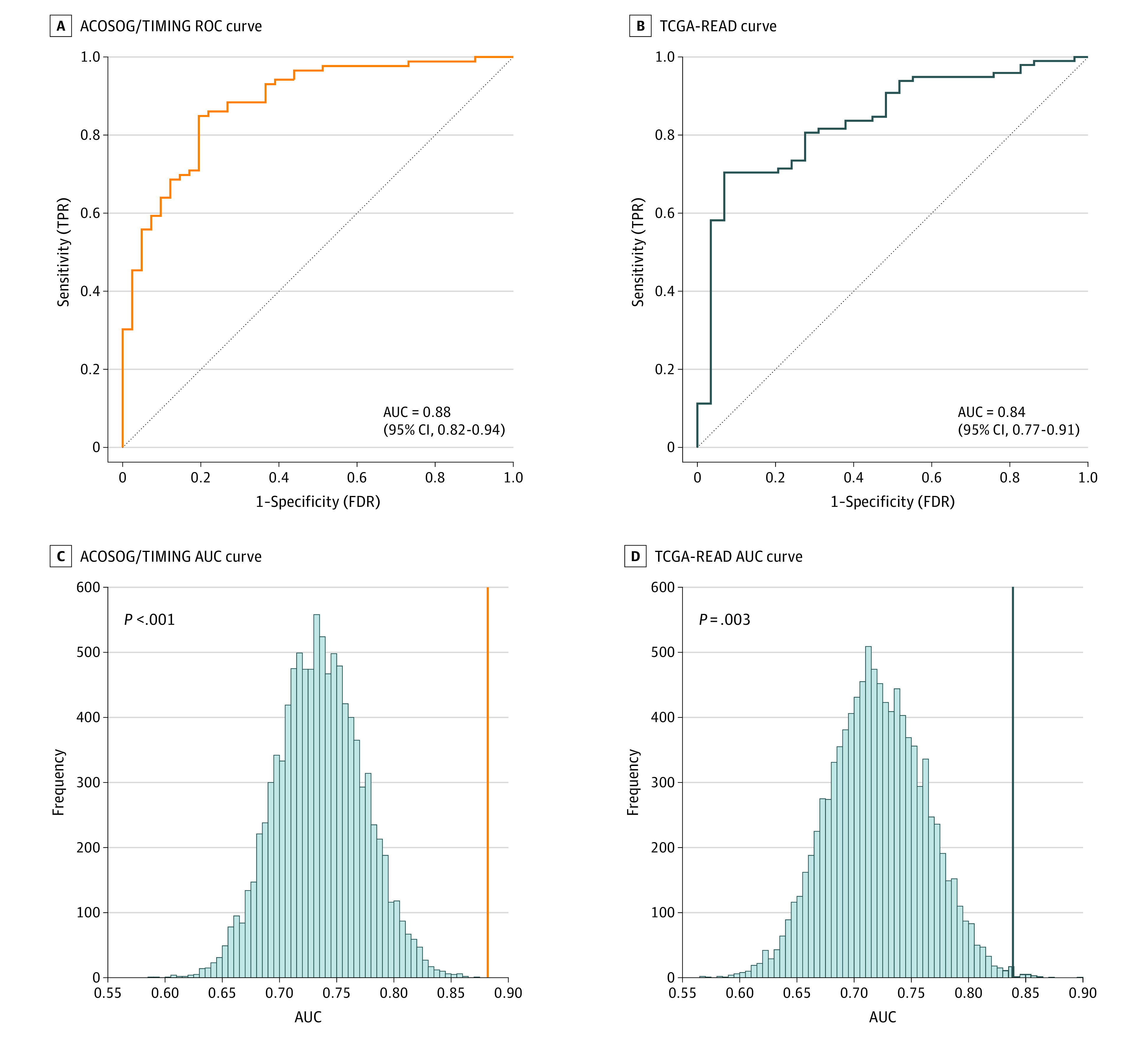
Diagnostic Accuracy of the 19–MicroRNA (miRNA) Signature in Differentiating Stage I vs Stage II or III Rectal Cancers A and B, Receiver operating characteristic (ROC) curves of the American College of Surgeons Oncology Group (ACOSOG)/Timing of Rectal Cancer Response to Chemoradiation (TIMING) and The Cancer Genome Atlas Rectum Adenocarcinoma (TCGA-READ) cohorts comparing the diagnostic ability of the 19-miRNA signature to distinguish stage I from stage II or III rectal tumors. C and D, The distributions of area under the curve (AUC) values computed after 10 000 random permutations are shown after disconnecting the link between miRNA expression and the associated clinical stage in the ACOSOG/TIMING and TCGA-READ cohorts. FDR indicates false discovery rate; TPR, true positive rate.

We assessed the discriminative power of the 19 differentially expressed miRNAs to distinguish the clinical stage of rectal tumors. We built a binary classifier using a logistic regression model that used the levels of expression of these miRNAs as inputs. We then drew ROC curves and computed the AUCs (Figure 2A). We also drew calibration curves and computed the Brier score associated with each model (eFigure 2 in the Supplement). The AUCs measured for distinguishing stage I from stage II or III rectal cancers with the classifiers built using our proposed miRNA signature were 0.88 (95% CI, 0.83-0.94) for ACOSOG/TIMING and 0.84 (95% CI, 0.77-0.91) for TCGA-READ. We also performed a random permutation experiment in which we relearned the coefficients for the 19-miRNA binary classifier after randomly shuffling the stage group labels. This finding corresponds to a null hypothesis of independence between the clinical-stage labels and the actual miRNA levels measured for each sample. The empirical distributions of AUC values computed under this null hypothesis after 10 000 random permutations are shown in Figure 2B. For ACOSOG/TIMING, the empirical *P* value associated with the AUC value of 0.88 that we had originally observed was *P* < .001, and the empirical *P* value associated with the AUC value of 0.84 was *P* = .003 for TCGA-READ. Therefore, we conclude that the discriminative power of our classifier is significantly better than the one that we would expect if there was no biological connection between the levels of expression of the selected miRNAs and clinical stage.

### Prediction of the Functional Interaction of miRNA With Target Genes

**Figure 3.**
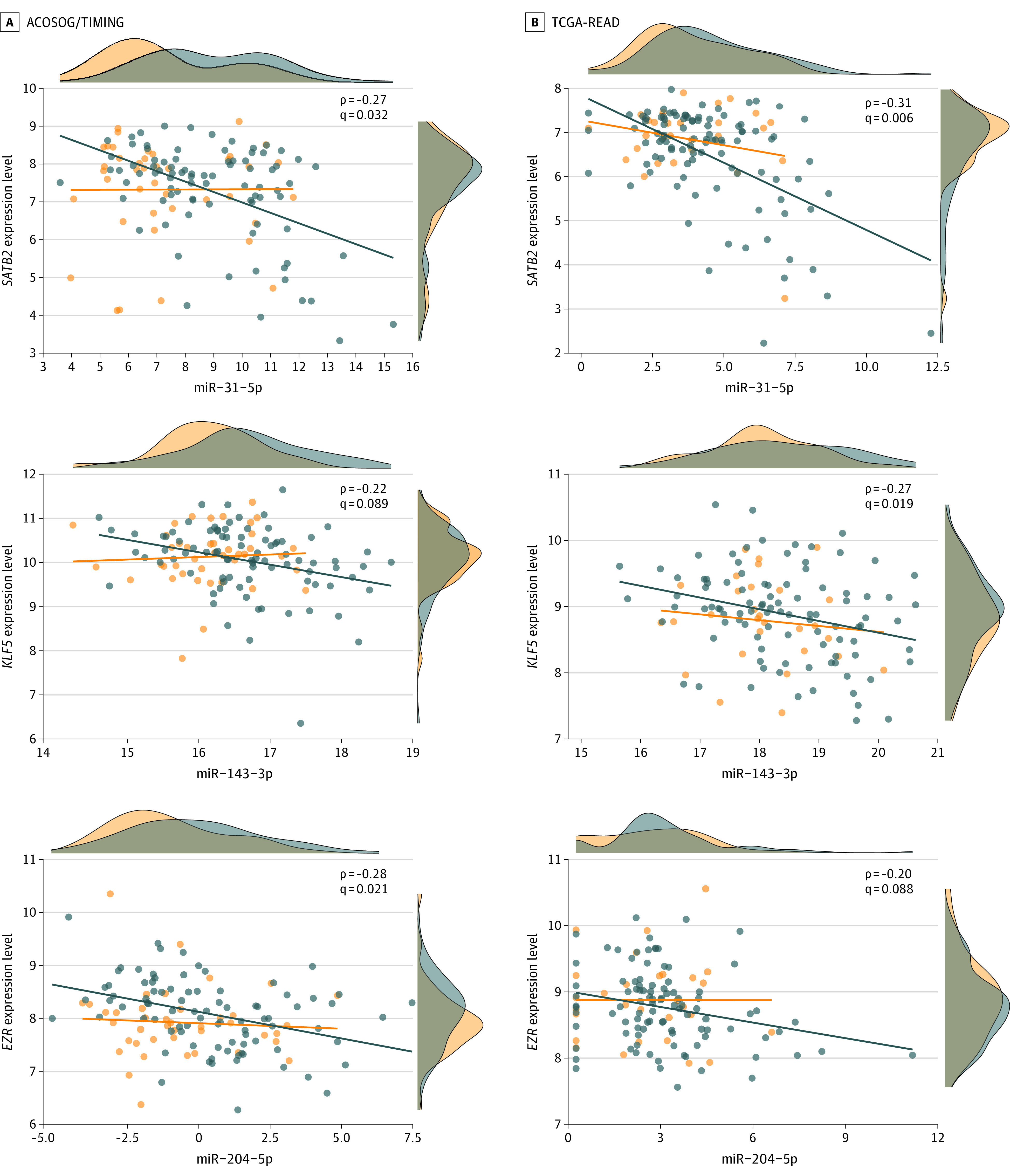
Correlation of the MicroRNAs (miRNAs) miR-31-5p, miR-143-3p, and miR-204-5p With Their Target Genes in 2 Independent Cohorts The expression levels of miR-31-5p and its target gene *SATB2* has a negative Spearman correlation (ρ < 0, false discovery rate < 0.1) in both the American College of Surgeons Oncology Group (ACOSOG)/Timing of Rectal Cancer Response to Chemoradiation (TIMING) and The Cancer Genome Atlas Rectum Adenocarcinoma (TCGA-READ) cohorts. miR-143-3p and *KLF5* as well as miR-204-5p and *EZR* exhibit inverse associations. Each dot represents the normalized expression level of the sample. Density plots on the x and y axes represent the distribution of the miRNA and its target gene expression values.

To explore the functional roles of these miRNAs, we identified known miRNA-mRNA interactions using miRTarBase, a database supported by evidence from functional studies such as Western blot, quantitative polymerase chain reaction, or reporter assays.^23^ In this database, we identified 508 potential gene targets from 17 of the 19 miRNAs (eTable 6 in the Supplement). Because we expect the expression levels of mRNA and miRNA to be inversely correlated if they have a functional interaction, we integrated the mRNA profiling data and performed a Spearman correlation analysis (ρ < 0, FDR < 0.1) in respect to the expression level of miRNA in both the ACOSOG/TIMING and TCGA-READ cohorts. Following this analysis, we identified 12 miRNA-mRNA pairs in the ACOSOG/TIMING cohort and 47 miRNA-mRNA pairs in the TCGA-READ cohort (eTables 7 and 8 in the Supplement). In these 2 cohorts, we found that 3 miRNA-mRNA pairs exhibited consistent associations: miR-31-5p-*SATB2* (OMIM 612313), miR-143-3p-*KLF5* (OMIM 602903), and miR-204-5p-*EZR* (OMIM 123900). All these miRNAs were higher in stage II or III vs I tumors and were inversely correlated with the expression of their target genes (Figure 3; eTables 7 and 8 in the Supplement).

## Discussion

In this diagnostic study, we investigated miRNA-mRNA interactions that may be associated with tumor progression by performing an integrative analysis of miRNA and mRNA in 2 independent cohorts of stage I vs stage II or III rectal cancers. We found 19 miRNAs that were differentially expressed by the clinical stage in both cohorts and found this signature to be excellent at distinguishing stage I from stage II or III rectal cancers. By integrating mRNA data into the analysis, we found 3 miRNA-mRNA pairs (miR-31-5p-*SATB2*, miR-143-3p-*KLF5*, and miR-204-5p-*EZR*) that exhibited consistent patterns in both cohorts.

Several studies^24,25,26,27^ have found that miRNA expression in patients with colorectal cancer is dysregulated compared with healthy individuals. Of interest, several of the 19 that we identified in our analysis are dysregulated in colorectal cancer.^24,25,26,27,28,29^ Some of these miRNAs are even detected in the serum and can potentially serve as noninvasive biomarkers to diagnose colorectal cancer.^25,26^ Our findings of the differential expression of these important miRNAs by the TNM stage of the tumor add further clinical significance to these miRNAs. Specifically in rectal cancer, this could have clinical utility because differentiation of stage I from stage II or III rectal cancers is not always apparent on standard workup. Supplementation of the clinical data with a distinguishing biomarker could prove to be helpful in selecting the appropriate treatment and determining prognosis.

Apart from potentially improving diagnosis, understanding the functional roles of these miRNAs could also uncover novel therapeutic targets. miR-31-5p has been reported to be overexpressed in more advanced–stage rectal cancers, which is consistent with our findings.^29^ miR-31-3p, the complementary strand of miR-31-5p, is associated with benefit from anti–epidermal growth factor receptor therapy in certain patients.^30,31^ *SATB2*, the predicted gene target of miR-31-5p in our analysis, is known to be associated with chromatin remodeling and regulating gene expression.^32^ In a prospective cohort of patients with colorectal cancer, lower expression of *SATB2* in the immunohistochemical staining of the tumor was associated with worse survival.^33^ Cell line and in vivo experiments have found that miR-31 represses *SATB2* to promote carcinogenesis and tumor invasion in colorectal cancer.^34^ This finding validates and explains the functional association of miR-31-5p-*SATB2* that we identified in our integrative analysis. miR-143-5p, which was overexpressed in stage II or III vs stage I tumors in our analysis, has also been associated with more advanced disease and worse survival.^35,36,37^ On the contrary, miR-143-3p expression levels have been reported to be diminished in more advanced tumors.^36^ In colorectal cancer cell lines, overexpression of miR-143 downregulates *KLF5*.^38^ *KLF5* is a transcription factor that is predominantly expressed in the intestinal crypt and regulates cell proliferation.^39^ It has also been implicated as an essential mediator of tumorigenesis by interacting with the *RAS* and Wnt signaling pathways.^40,41^ miR-204-5p has been reported to be a tumor suppressor^42^ and downregulates the expression of *EZR* in cell line studies.^43^ *EZR* organizes the microvilli^44^ and pathologically is implicated in tumor invasion and metastasis.^45,46^

Many of the miRNAs that are implicated as having tumor suppressive properties were paradoxically overexpressed in more advanced tumors. The members of the let-7 family are thought to serve as tumor suppressors by inhibiting cell proliferation and invasion and have been extensively reported to be dysregulated in colorectal cancer.^25,26,28,47^ miR-99a-5p and miR-100-3p inhibit mTOR signaling.^48^ miR-143-3p and miR 214 inhibit colorectal liver metastases.^49,50^ miR-34c and miR-129 inhibit tumor proliferation in vitro.^51,52^ miR-218-5p inhibits angiogenesis and epithelial-mesenchymal transition of the tumor.^53^ The contribution of these miRNAs to carcinogenesis and tumor progression is still not fully comprehended and warrants further investigation.

### Limitations

Our study has limitations. Although there are many available tools to predict the gene targets of miRNA,^54^ the results from these databases are not always consistent. We chose to predict the miRNA gene targets using miRTarBase because this database curates experimentally validated miRNA-mRNA interactions.^23^ However, given this requirement for mechanistic evidence, we may have missed potentially relevant interactions in our analysis. Furthermore, we filtered the interactions by negatively correlating the expression of miRNA and its target gene. However, we may have inadvertently filtered out some true interactions with this approach because the network of miRNA-mRNA interactions is much more complex. We also attempted correlative analyses of miRNA expression with clinical outcomes, such as pathological complete response and survival, but did not observe meaningful patterns, likely because of the small sample size.

## Conclusions

This study identified 19 miRNAs that were differentially expressed in stage I and stage II or III rectal cancers that could serve as diagnostic biomarkers. Furthermore, the interactions of these miRNAs with their target genes may be associated with the progression of rectal cancer. miR-31-5p has been previously reported to regulate colorectal cancer progression by repressing *SATB2*. The roles of miR-143-3p-*KLF5* and miR-204-5p-*EZR* have not been characterized before; therefore, these are good candidates for future functional studies.

## Notes

Supplement. **eTable 1.** Clinicopathologic Characteristics of the ACOSOG/TIMING Cohort **eTable 2.** Normalized miRNA Data of ACOSOG/TIMING Cohort **eTable 3.** Differentially Expressed miRNA in ACOSOG/TIMING Cohort **eTable 4.** Normalized miRNA Data of TCGA-READ Cohort **eTable 5.** Differentially Expressed miRNA in TCGA-READ Cohort **eTable 6.** Predicted Gene Targets of the 19 Differentially Expressed miRNA by miRTarBase **eTable 7.** miRNA-mRNA Pairs in ACOSOG/TIMING Cohort After Filtering by SPEARMAN Correlation **eTable 8.** miRNA-mRNA Pairs in TCGA-READ Cohort After Filtering by SPEARMAN Correlation **eFigure 1.** Analysis of Differences in Age and Tumor Cellularity Across Stage Groups in Rectal Cancer Patients **eFigure 2.** Calibration Curves and Predictive Accuracy Measures for the Logistic Regression Models
